# eEF1α2 is required for actin cytoskeleton homeostasis in the aging muscle

**DOI:** 10.1242/dmm.050729

**Published:** 2024-08-29

**Authors:** Hidetaka Katow, Hyung Don Ryoo

**Affiliations:** Department of Cell Biology, New York University Grossman School of Medicine, New York, NY 10016, USA

**Keywords:** eEF1α2, *Drosophila*, Muscle, Aging, Actin, Myosin

## Abstract

The translation elongation factor eEF1α (eukaryotic elongation factor 1α) mediates mRNA translation by delivering aminoacyl-tRNAs to ribosomes. eEF1α also has other reported roles, including the regulation of actin dynamics. However, these distinct roles of eEF1α are often challenging to uncouple and remain poorly understood in aging metazoan tissues. The genomes of mammals and *Drosophila* encode two eEF1α paralogs, with eEF1α1 expressed ubiquitously and eEF1α2 expression more limited to neurons and muscle cells. Here, we report that *eEF1α2* plays a unique role in maintaining myofibril homeostasis during aging in *Drosophila*. Specifically, we generated an *eEF1α2* null allele, which was viable and showed two distinct muscle phenotypes. In young flies, the mutants had thinner myofibrils in indirect flight muscles that could be rescued by expressing *eEF1α1*. With aging, the muscles of the mutant flies began showing abnormal distribution of actin and myosin in muscles, but without a change in actin and myosin protein levels. This age-related phenotype could not be rescued by *eEF1α1* overexpression. These findings support an unconventional role of *Drosophila* eEF1α2 in age-related homeostasis of muscle myofibers*.*

## Teaser

**Summary:** *Drosophila* eEF1α2, a well-characterized translation elongation factor, is required for age-related muscle fiber homeostasis, possibly independent of its role in protein synthesis.

## INTRODUCTION

Cellular mRNA translation occurs through the cooperative action of many initiation, elongation and termination factors (Hershey et al., 2019). Some mRNA translation regulators reportedly have other non-canonical activities unrelated to protein synthesis. However, these alternative functions are often difficult to study through genetics, as mutations that globally impair mRNA translation can cause cell lethality.

Among the translation regulators with non-canonical functions include eukaryotic elongation factor 1 α (eEF1α). eEF1α is best characterized as a translation elongation factor that delivers aminoacyl-tRNAs to the A site of ribosomes (Andersen et al., 2003; Voorhees and Ramakrishnan, 2013). Many metazoan species have two eEF1α paralogs, referred to as eEF1α1 and eEF1α2. These two paralogs share 95% similarity in protein sequence and 82% similarity in gene sequence. In mammals, eEF1α1 is ubiquitously expressed except for the muscle tissues and plays essential roles in protein synthesis (Kahns et al., 1998; Soares et al., 2009). In contrast, mammalian eEF1α2 expression occurs specifically in the muscle and neurons during early embryonic to postnatal development (Griffiths et al., 2012; Doig et al., 2013; Davies et al., 2023). Publicly available single-cell RNA-sequencing data show that *Drosophila* eEF1α2 expression is similarly specific to neurons, muscle cells and a few male germline cells (FlyBase: https://flybase.org/reports/FBgn0000557#expression). Reflecting its limited role, the loss of eEF1α2 in mammals does not cause developmental lethality. For example, eEF1α2 mutant alleles have been found in patients with autism and epilepsy (Nakajima et al., 2015; Lam et al., 2016; Carvill et al., 2020; Mohamed and Klann, 2023). In addition, eEF1α2 is deleted in a mouse line referred to as *wasted* (*wst*) (Chambers et al., 1998). Homozygous mutant *wasted* mice show motor neuron degeneration (Lutsep and Rodriguez, 1989; Newbery et al., 2005), loss of muscle bulk and death by 4 weeks (Tezuka et al., 1986; Newbery et al., 2007).

Whether the phenotypes associated with eEF1α2 loss in mammals can be entirely attributed to reduced protein synthesis remains unclear. This is because eEF1α proteins have a number of ‘moonlighting’ functions (Lukash et al., 2004; Matsuda et al., 2004; Sasikumar et al., 2012; Abbas et al., 2015; Losada et al., 2018). For example, eEF1α reportedly binds and inhibits general control nonderepressible protein 2 (GCN2), a signaling kinase activated upon amino acid deprivation (Visweswaraiah et al., 2011; Silva et al., 2016; Ramesh and Sattlegger, 2020). In addition, eEF1α can bind and regulate the actin cytoskeleton in yeast and cultured mammalian neurons (Gross and Kinzy, 2005; Mendoza et al., 2021). *In vitro*, both eEF1α1 and eEF1α2 are able to bind actin, and form dimers to bring actin filaments together and promote actin bundle formation (Amiri et al., 2007; Morita et al., 2008; Vlasenko et al., 2015; Negrutskii et al., 2022; Mohamed and Klann, 2023). However, uncoupling the moonlighting roles of eEF1α2 from its role in protein synthesis in metazoan tissues has remained challenging. Moreover, *in vitro* and cell culture experiments have been inadequate in uncovering possible roles of eEF1α2 during aging.

In this study, we used the facile tools of *Drosophila* genetics to examine the *in vivo* roles of eEF1α2 during aging. *eEF1α2* mutants generated by CRISPR/Cas9 gene editing survived to adulthood but showed degenerative phenotypes in the muscle and photoreceptors during aging. In the muscle, the loss of *eEF1α2* led to an age-related defect in actin and Myosin heavy chain (Mhc) distribution. Interestingly, such decline was neither associated with changes in actin and Mhc protein levels during aging nor rescued by the *eEF1α2* paralog, *eEF1α1*. These results support the role of eEF1α2 in age-related protein homeostasis, possibly independent of its role in protein synthesis.

## RESULT

### The CRISPR gene-edited *Drosophila eEF1α2* mutant exhibits abnormal wing postures

**Fig. 1:**
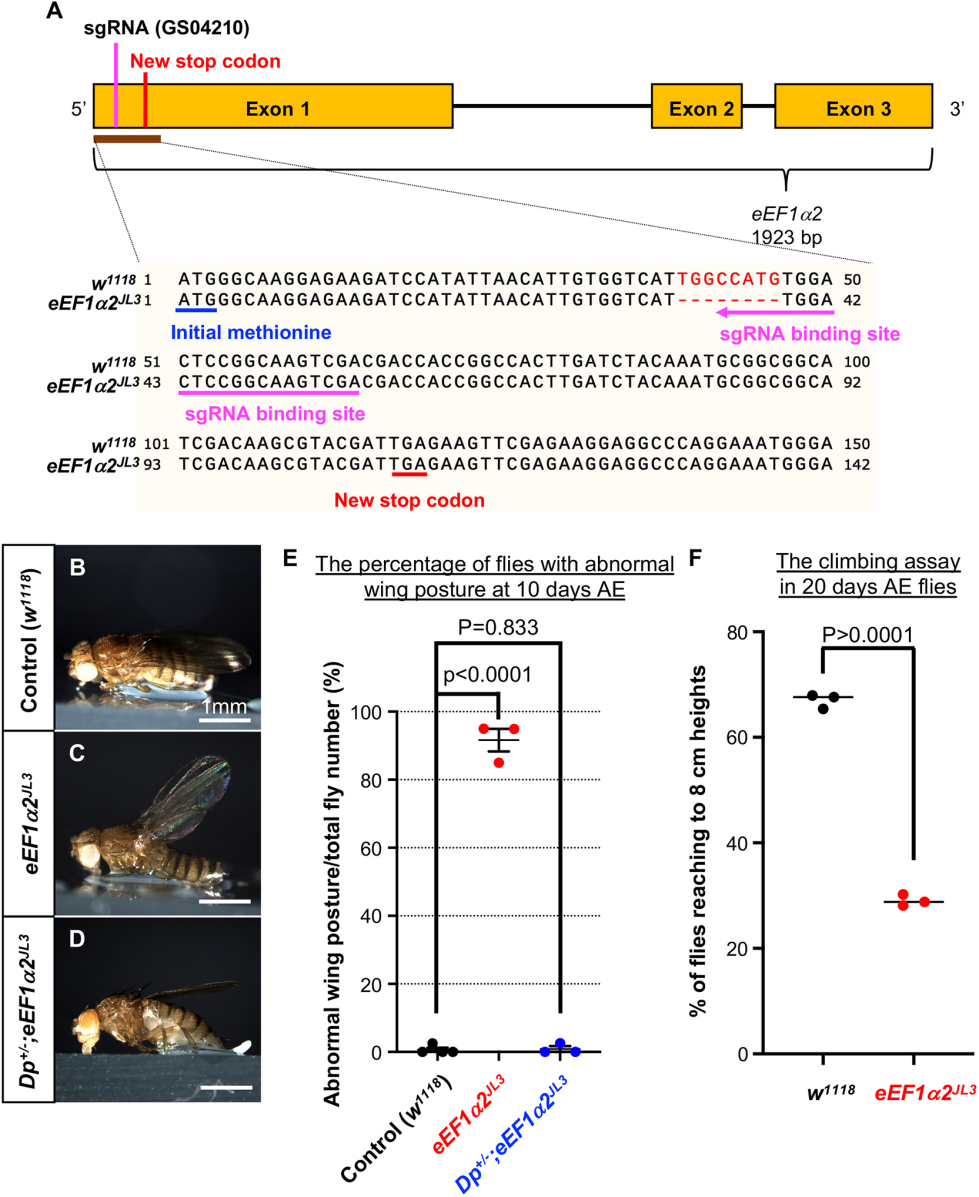
**. An *eEF1α2* null mutant allele generated through CRISPR/Cas9 gene editing shows age-dependent phenotypes.** (A) A schematic of the *eEF1α2^JL3^* coding region. The sgRNA line GS04210 targets a region 45 bp away from the AUG start codon (blue line). The *eEF1α2^JL3^* allele has an 8 bp deletion that generates a premature stop codon (red line) within exon 1. (B-D) Representative images of adult flies with distinct wing postures. Control flies (genotype *w^1118^*) have wings stretched horizontally (B). The *eEF1α2^JL3^* homozygous mutant has vertically stretched wings (C). Introducing a genomic duplication with *eEF1α2* into the *eEF1α2^JL3^* homozygous background (genotype, *Dp^+/−^;eEF1α2^JL3^*) rescues the wing phenotype (D). (E) Quantification of the abnormal wing posture phenotype in B-D. For each genotype, 31-56 flies per vial were examined for wing posture defects. The percentages of flies with abnormal wing posture in each vial (*n*=the number of vials examined) are shown. *w^1118^*, *n*=4; *eEF1α2^JL3^*, *n*=3; *Dp^+/−^;eEF1α2^JL3^*, *n*=3. *P*-values were calculated using unpaired two-tailed *t*-test. Bars show mean±s.e.m. (F) The locomotor capabilities of flies 20 days after ecclosion (AE) were assessed through climbing assays. Flies were tapped to the bottom of the vial and the percentage of flies that climbed up to the 8 cm high mark within 20 s was counted. The plot is a result of three independent trials, with the following number of flies in each vial: *w^1118^* (vial 1, *n*=28; vial 2, *n*=30; vial 3, *n*=29) and *eEF1α2^JL3^* (vial 1, *n*=20; vial 2, *n*=25; vial 3, *n*=28). *P*-value was calculated by unpaired two-tailed *t*-test. Bars show median values.

The *Drosophila melanogaster* genome encodes two *eEF1α* paralogs, *eEF1α1* and *eEF1α2*. Using *eEF1α2*-targeting gRNA transgenes (GS04210; Hu et al., 2017) (Fig. 1A) and maternal Cas9, we generated several fly lines with small deletions within the coding sequence (Fig. 1A, hyphens). These deletions introduced new stop codons (Fig. 1A, red underline) downstream of the gRNA targeting region (Fig. 1A, magenta underline). We selected one of these loss-of-function mutants (*eEF1α2^JL3^*) for further analysis.

More than 90% of the homozygous *eEF1α2^JL3^* mutants reached adulthood, but with a slight developmental delay (Fig. S1A). There was no significant body weight difference between the newly eclosed *eEF1α2^JL3^* adults and the control wild-type flies (Fig. S1B). We noted that, as the flies began to age, the mutant flies began showing abnormal wing postures. Specifically, the wild-type flies had wings stretched horizontally close to their thorax (Fig. 1B), whereas the mutants had wings stretched at an abnormal angle (Fig. 1C). Most mutants displayed such abnormal wing postures within 10 days after eclosion (AE) (Fig. 1E). This phenotype was completely suppressed when a chromosome with *eEF1α2* gene duplication was introduced into the background of the *eEF1α2^JL3^* mutant flies (*Dp^+/−^;eEF1α2^JL3^*) (Fig. 1D,E).

Correlating with the emergence of the age-related wing posture phenotype, we noted that old *eEF1α2^JL3^* flies showed reduced locomotor activity. We quantified this phenotype in flies 20 days AE by subjecting the mutant and control flies to climbing assays. Specifically, we collected more than 20 flies from each genotype and counted the number of flies that could climb above the 8 cm mark within 20 s after being tapped to the bottom. The *eEF1α2^JL3^* homozygous flies exhibited a statistically significant reduction in their climbing ability compared to that of the *w^1118^* controls (Fig. 1F). These data indicated that the mutants have age-related phenotypes affecting their wings and their locomotor function.

### *eEF1α2* is required for the maintenance of myofibril thickness in adult flight muscles

**Fig. 2.**
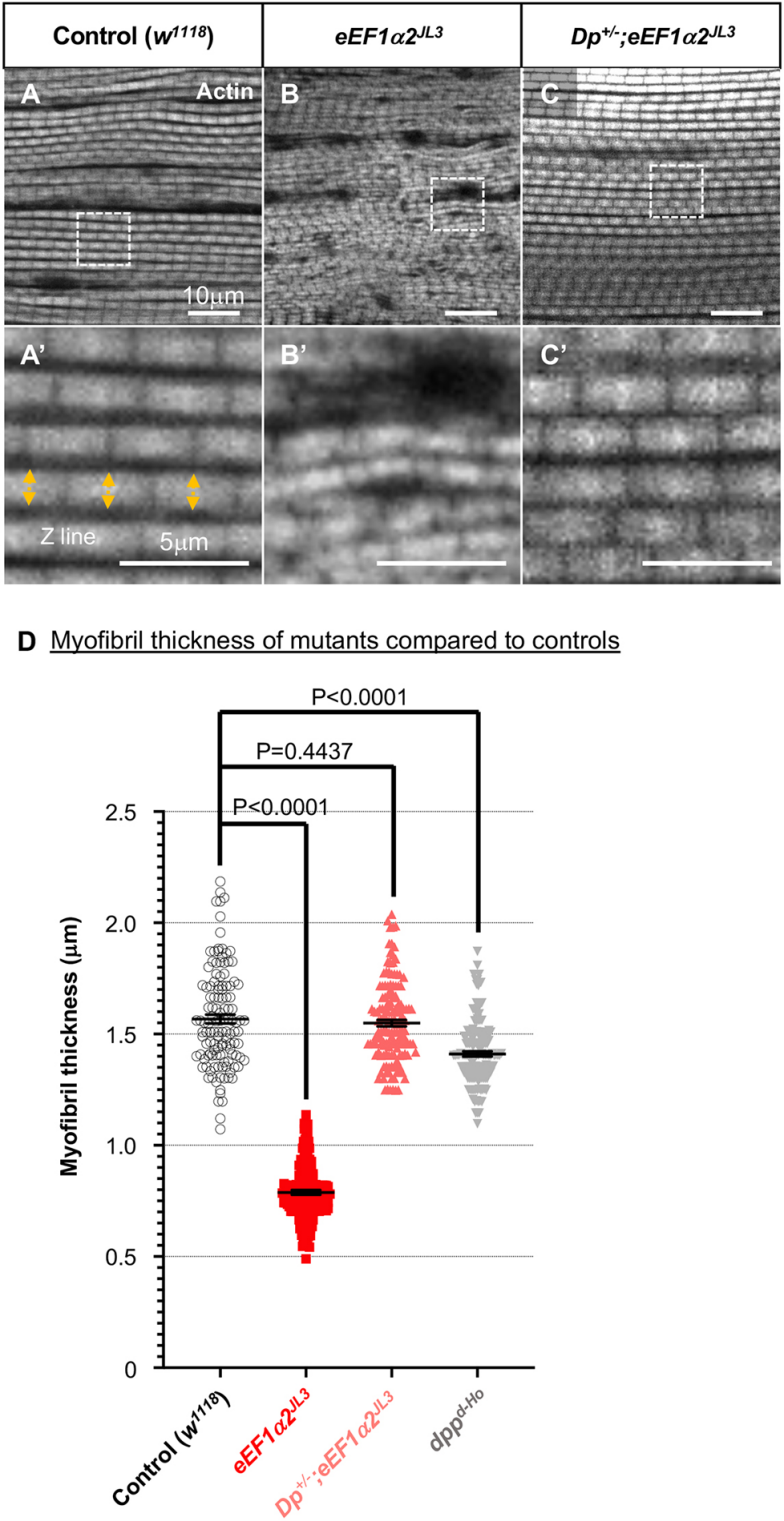
***eEF1α2^JL3^* homozygous flies have thinner myofibrils in the indirect flight muscles.** (A-C) F-actin, visualized with phalloidin labeling (grayscale), in indirect flight muscles (IFMs) of 0-day-old adult flies. (A′-C′) Magnified images of the areas marked with white dashed squares in A-C. Gentoypes: control *w^1118^* (A,A′), *eEF1α2^JL3^* (B,B′) and *Dp^+/−^;eEF1α2^JL3^* (C,C′). Arrows in A′ show individual myofibril thickness, which was quantified in D. (D) For quantification of myofibril thickness, five continuous sarcomeres were chosen from five different myofibrils from three to five individual flies. *w^1118^*, *n*=125; *eEF1α2^JL3^*, *n*=150; *Dp^+/−^;eEF1α2^JL3^*, *n*=150; *dpp^d-Ho^*, *n*=150; where *n* is the number of sarcomeres examined. *P*-values were calculated using unpaired two-tailed *t*-tests. Error bars show mean±s.e.m. The control *w^1118^* and *eEF1α2^JL3^* data are also shown in Fig. 5D for comparison with data from flies expressing eEF1α1 in the *eEF1α2^JL3^* background.

Because the decline in skeletal muscle function could affect both the wing posture and the locomotor activity, we examined the structure of muscle myofibrils of the indirect flight muscles (IFMs) that control wing movement (Johnston et al., 2020). We first visualized the F-actin fibers in the muscles using phalloidin labeling (Fig. 2A-Cʹ), which revealed clear outlines of each sarcomere in the myofibrils. The stained IFMs from flies 0 day AE revealed that the *eEF1α2^JL3^* mutant had myofibrils with approximately half the thickness of that in the *eEF1α2* wild-type controls (*w^1118^*) (Fig. 2D). The impact of *eEF1α2* loss on the myofibril structure was specific to adults, as the *eEF1α2^JL3^* mutant larvae did not exhibit abnormalities in muscle myofibril thickness (Fig. S2A,B). The adult myofibril thickness phenotype associated with *eEF1α2* loss was rescued when the *eEF1α2* gene duplication chromosome was introduced into the background of *eEF1α2^JL3^* flies (Fig. 2C,C′,D). These findings indicate that *eEF1α2* acts specifically in adult muscles, playing a positive role in controlling myofibril size.

One of the well-described *Drosophila* mutants with abnormal wing posture is the *decapentaplegic* (*dpp*) mutant *dpp^d-Ho^* (Spencer et al., 1982; Gelbart, 1989). Dpp is a TGF-β family ligand that regulates diverse aspects of *Drosophila* development. We compared the phenotypes of the *eEF1α2^JL3^* and *dpp^d-Ho^* mutants. We noted that some *eEF1α2^JL3^* mutants displayed wing angles maintained at 90° vertically and others downwards. By contrast, *dpp^d-Ho^* mutants had wings angled horizontally at 90° (Fig. S3A,B). The myofibril thickness of *dpp^d-Ho^* flies was similar to that of the wild-type control flies on day 0 AE (Fig. 2D). These results indicate that the wing phenotypes and myofibril structure resulting from a lack of *eEF1α2* are distinct from those of the classic *dpp^d-Ho^* mutant that affects wing posture.

### The *eEF1α2^JL3^* mutant shows age-dependent disorganization of muscle actin fibers

**Fig. 3.**
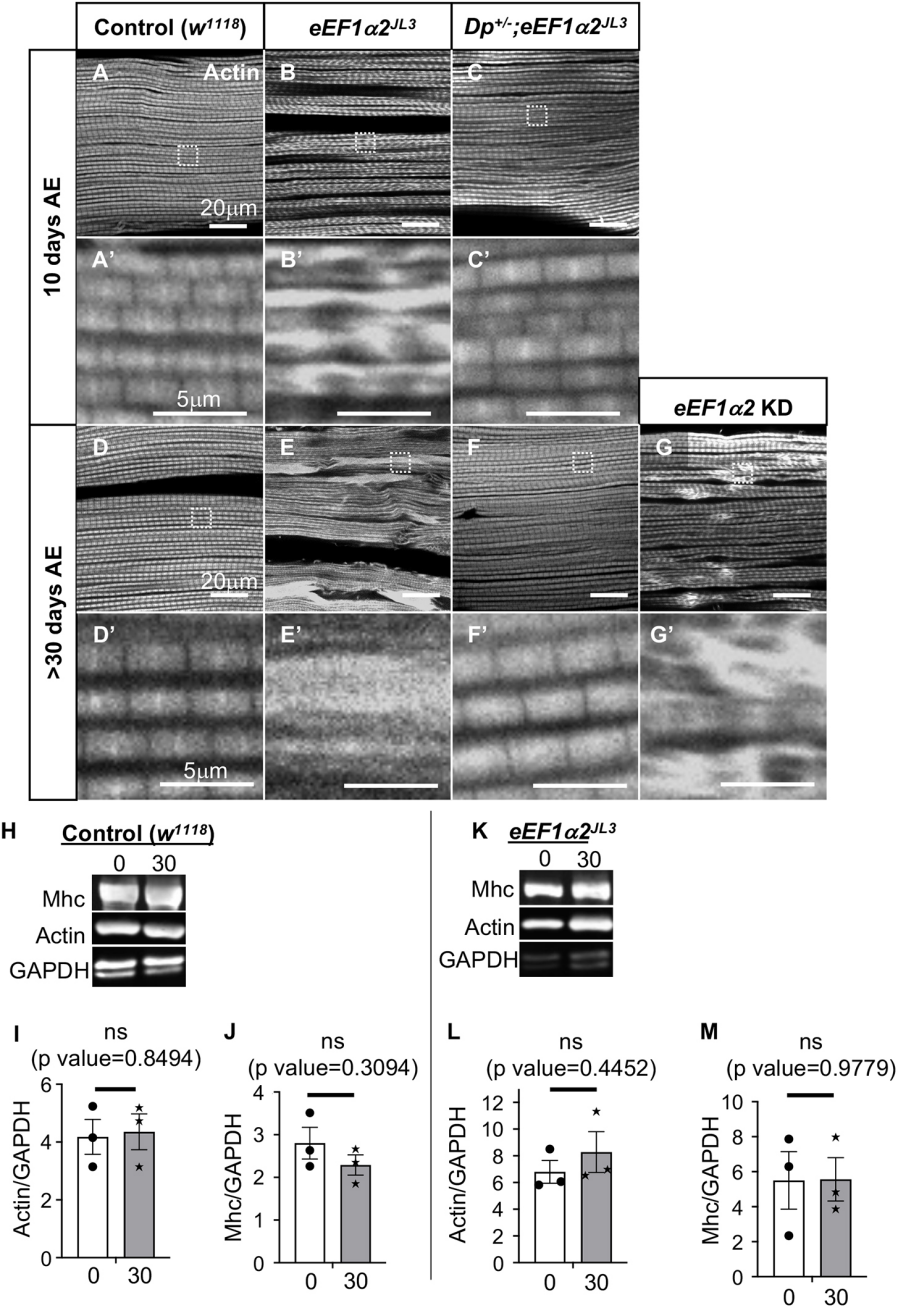
**Uneven actin distribution in the muscles of old *eEF1α2^JL3^* mutant flies.** (A-Cʹ) F-actin visualized with phalloidin labeling (grayscale) in 10-day-old fly IFMs. Areas marked with white dashed lines in A-C are enlarged in A′-C′. Genotypes: control *w^1118^* (A,A′), *eEF1α2^JL3^* (B,B′), *Dp^+/−^;eEF1α2^JL3^* (C,C′). (D-Gʹ) Actin (grayscale) distributions in IFMs of flies more than 30 days old. White dashed squares in D-G are enlarged in D′-G′. Genotypes: control *w^1118^* (D,D′), *eEF1α2^JL3^* (E,E′), *Dp^+/−^;eEF1α2^JL3^* (F,Fʹ), *Act88F-gal4/ uas-eEF1α2 RNAi;uas-dicer2/+* (G,Gʹ). Note the abnormal distribution of actin in *eEF1α2^JL3^* mutants (E,Eʹ). The re-introduction of *eEF1α2* through the *Dp* restores actin distribution in *eEF1α2^JL3^* homozygous mutant IFMs (F,Fʹ). An IFM from flies with muscle-specific *eEF1α2* RNAi is shown (G,G′). *Uas-dicer2* was combined for a more efficient knockdown (KD). (H-M) Western blot analyses of the indicated proteins from extracts of 0-day-AE and more than 30-day-AE fly IFMs. Samples were prepared from equal numbers of flies. (H) Blots from control *w^1118^* flies. (I,J) Quantification of relative actin (I) and Mhc (J) levels from control flies, normalized to GAPDH levels. Error bars show mean±s.d. Unpaired two-tailed *t*-test was used to assess statistical significance. (K) Comparison of the actin and Mhc protein levels between 0-day-AE and more than 30-day-AE *eEF1α2^JL3^* homozygous IFM extracts. (L,M) Quantification of relative actin (L) and Mhc (M) levels from *eEF1α2^JL3^* flies, normalized to GAPDH levels. Unpaired two-tailed *t*-test was used to assess statistical significance. Error bars show mean±s.e.m. ns, not significant.

Although the young mutant flies had thinner muscle fibers, these flies did not necessarily exhibit the abnormal wing posture phenotype. Therefore, we examined the IFMs in older flies with externally visible wing posture defects. We found uneven distribution of actin fibers in IFMs of 10-day-old *eEF1α2^JL3^* adults (Fig. 3B,B′) but not in control flies (Fig. 3A,A′). Specifically, there were subregions of intense actin enrichment found in the *eEF1α2^JL3^* mutant (Fig. 3B,B′). At 30 days AE, the extent of the uneven actin distribution became more pronounced (Fig. 3E,E′) compared to that in young flies (Fig. 3B,B′) or age-matched control flies (Fig. 3D,D′). The actin distribution phenotype was completely rescued by the introduction of an *eEF1α2* duplication (*Dp^+/−^;eEF1α2^JL3^*; Fig. 3C,C′,F,F′), further validating that the abnormal actin distribution was due to the loss of *eEF1α2*. Moreover, IFM-specific *eEF1α2* knockdown also showed uneven actin distributions in flies more than 30 days old (Fig. 3G,G′). These results indicate that the loss of *eEF1α2* impairs proper actin distribution, specifically in the aging *Drosophila* myofibrils.

eEF1α is best known for its role in protein synthesis, but there were no significant changes in the protein levels of actin and GAPDH (used as a control, top band) between extracts prepared from equal numbers of 0- and 30-day-AE flies (Fig. 3H,I,K,L). Because actin levels do not correlate with age-related changes in actin distribution of *eEF1α2* mutants, the results suggest a translation-independent role of eEF1α2 in myofiber homeostasis.

### Myosin distribution is moderately disrupted in *eEF1α2^JL3^* mutants

**Fig. 4.**
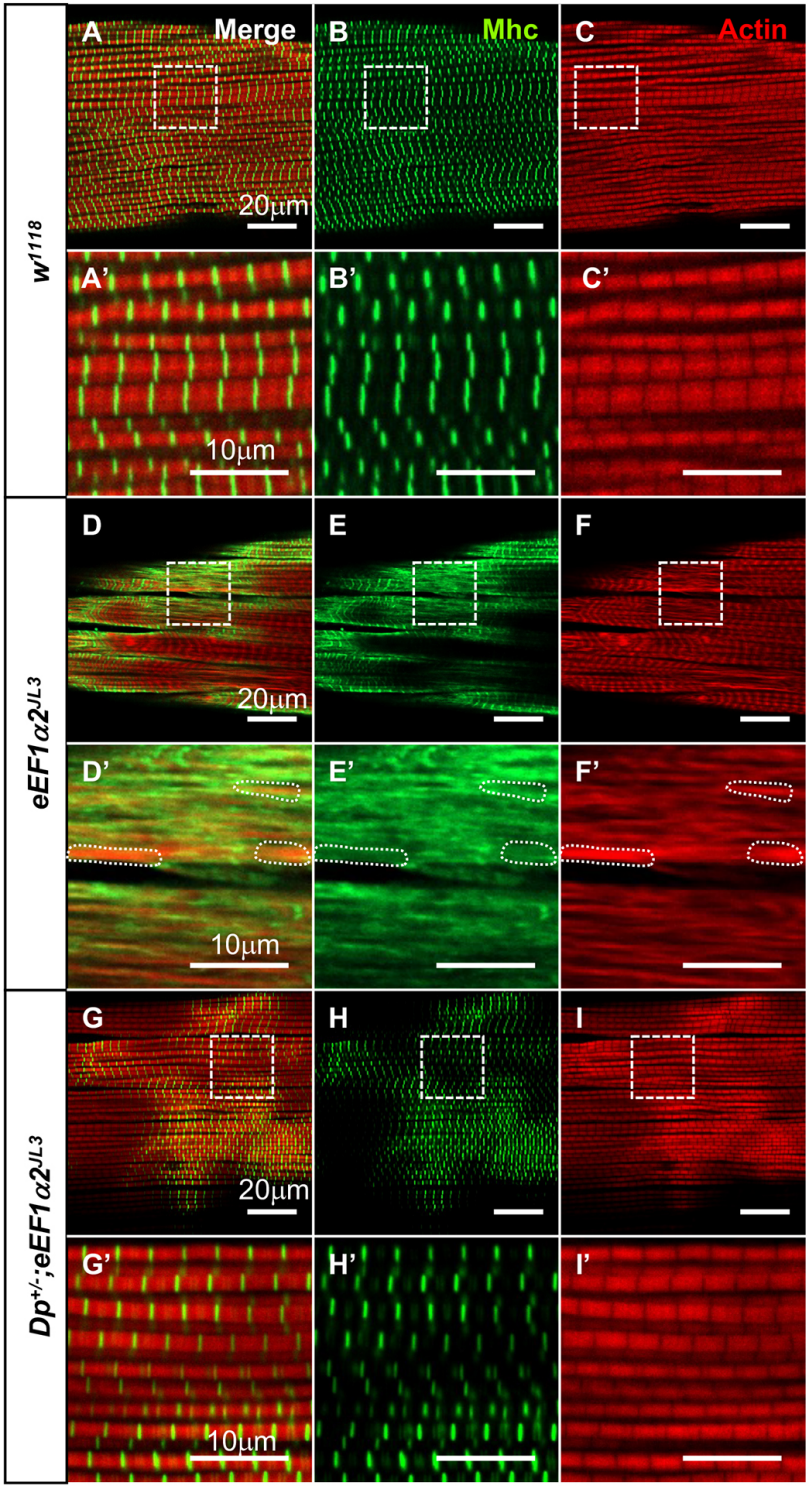
**Abnormal Mhc distribution in aged *eEF1α2^JL3^* mutant IFMs.** (A-I′) Actin (red) and Mhc (green) localization in fly IFMs more than 30 days old. The white dashed squares in A-I are magnified in A′-I′. (A-F) Representative images from a control *w^1118^* (A-C), *eEF1α2^JL3^* (D-F) and *Dp^+/−^;eEF1α2^JL3^* (G-I) IFMs are shown. The areas with uneven actin distributions are marked with white dashed regions (D′-F′). Images are representative of six samples per genotype.

The capability of the muscles to generate contractile force originates from the activity of the molecular motor myosin on its substrate actin filaments. Given the close relationship of myosin with actin, we visualized Mhc distribution patterns using an anti-Mhc antibody in IFMs of flies older than 30 days AE. Confocal microscope imaging revealed a loss of clear alignment of Mhc in *eEF1α2^JL3^* mutants (Fig. 4D-F′), unlike the well-organized Mhc patterns observed within the H zones in age-matched controls (Fig. 4A-C′). Although Mhc showed a more diffuse pattern in the mutants, we did not see enriched Mhc signals in the subregions with intense actin signals (Fig. 4D′-F′). Notably, the diffuse Mhc distribution phenotype of *eEF1α2^JL3^* mutants was rescued in *Dp^+/−^*;*eEF1α2^JL3^* flies (Fig. 4G-I′). The Mhc distribution change in the *eEF1α2^JL3^* mutant may be an indirect consequence of disrupted actin organization. Notably, a recent proteomic study identified 131 proteins that significantly interacted with human eEF1α2, but Mhc was not among them (Mendoza et al., 2021).

As with actin, Mhc protein levels did not change significantly with age in *eEF1α2^JL3^* mutant muscle extracts (Fig. 3H-M). These results imply a translation-independent role of eEF1α2 in the regulation of Mhc distribution.

### The overexpression of the eEF1α2 paralog eEF1α1 does not rescue the myofiber phenotypes in the aging IFM

Previous research has indicated that eEF1α1 and eEF1α2 have nearly identical functions, except for their affinity to GTP (Kahns et al., 1998). This finding prompted us to investigate the potential redundancy of eEF1α1 in the function of eEF1α2. To address this, we overexpressed *eEF1α1* in *eEF1α2* mutant IFMs. To do so, we used the IFM-specific *Act88F-gal4* driver (Gajewski and Schulz, 2010) to drive *UAS-eEF1α1* expression.

**Fig. 5.**
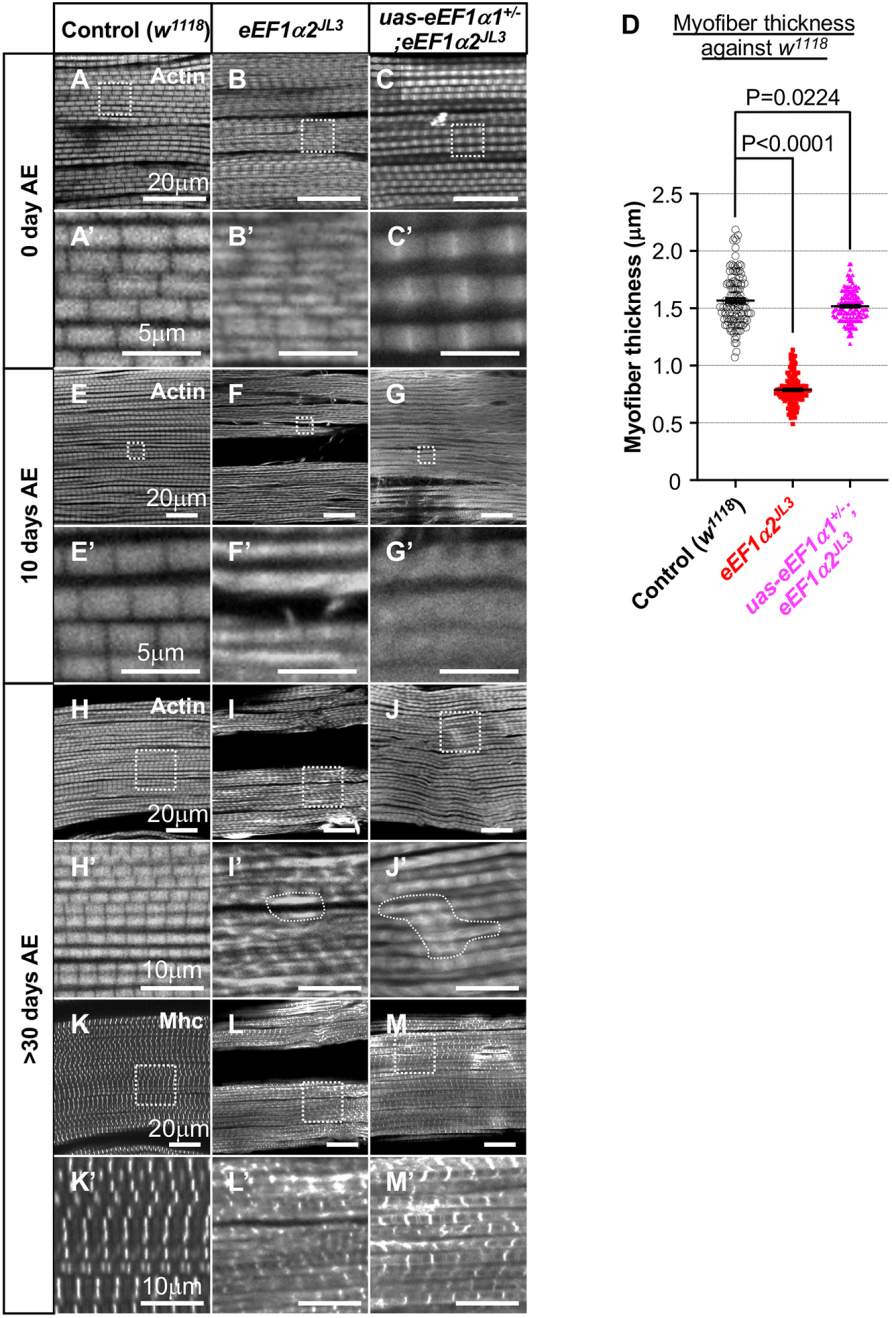
**The effect of overexpressing *eEF1α1* into the *eEF1α2^JL3^* background.** (A-Cʹ,E-Mʹ) Adult IFMs labeled with phalloidin (A-Cʹ,E-J′, grayscale) and anti-Mhc (K-M′, grayscale) in control *w^1118^* (A,Aʹ,E,Eʹ,H,Hʹ,K,Kʹ), *eEF1α2^JL3^* (B,Bʹ,F,Fʹ,I,Iʹ,L,Lʹ) and *eEF1α1* overexpression in the *eEF1α2^JL3^* background (C,Cʹ,G,Gʹ,J,Jʹ,M,Mʹ) are shown. Representative images are from 0-day-old (A-C′), 10-day-old (E-G′) and more than 30-day-old (H-M′) flies. The areas marked with white dashed squares in A-C,E-M are magnified in Aʹ-Cʹ,Eʹ-Mʹ. Areas of uneven actin distribution are surrounded by white dashed lines. Note that the control *w^1118^* and *eEF1α2^JL3^* IFM images are from identical genotypes as those shown in Fig. 2A,Aʹ and Fig. 4D-F, imaged under equivalent parameters. (D) Quantification of myofibril thickness in 0-day-AE flies. *w^1118^*, *n*=125; *eEF1α2^JL3^*, *n*=150; *uas-eEF1α1^+/−^;eEF1α2^JL3^*, *n*=150. Control (*w^1118^*) and *eEF1α2^JL3^* data (bars 1 and 2) are the same as those shown in Fig. 2D. Bar 3 shows that eEF1α1 overexpression can rescue the myofibril phenotype of *eEF1α2^JL3^*. Note that all myofibril thickness measurements were performed under equivalent conditions. *P*-values were calculated by unpaired two-tailed *t*-test. Error bars show mean±s.e.m.

First, we measured sarcomere thickness in the IFM of 0-day-AE flies (Fig. 5A-C′,D). We found that overexpression of *eEF1α1* in the *eEF1α2^JL3^* mutant IFMs restored the myofibril thickness (Fig. 5D) to a degree similar to that in the control flies. These results support the idea that *eEF1α1* and *eEF1α2* have shared functions in regulating myofibril thickness in young flies.

Next, we examined the consequences of *eEF1α1* overexpression throughout the lifespan of the mutant flies (Fig. 5A-C′,E-M′). In flies expressing *eEF1α1* in the *eEF1α2^JL3^* background, the uneven actin (Fig. 5E-J′) and Mhc (Fig. 5K-M′) distribution persisted at 10 days or more than 30 days AE. These results indicated that *eEF1α1* does not have redundant functions with *eEF1α2* in maintaining myofiber integrity in the aging flies.

### Trachea-ensheathing actin and myosin distributions appear in over 30-day-AE flies

In *Drosophila*, muscles are supplied with oxygen through the projection of the trachea. It is known that changes in tracheal morphology occur in hypoxia and tumor growths (Centanin et al., 2008; Mortimer and Moberg, 2009; Hsouna et al., 2010; Gervais and Bardin, 2021; Tamamouna et al., 2021). However, whether the muscle undergoes structural changes under disease conditions similar to those observed in the trachea remains unclear. Therefore, we investigated whether the muscle, especially the contacting regions projected by the trachea, exhibits abnormal structures.

**Fig. 6.**
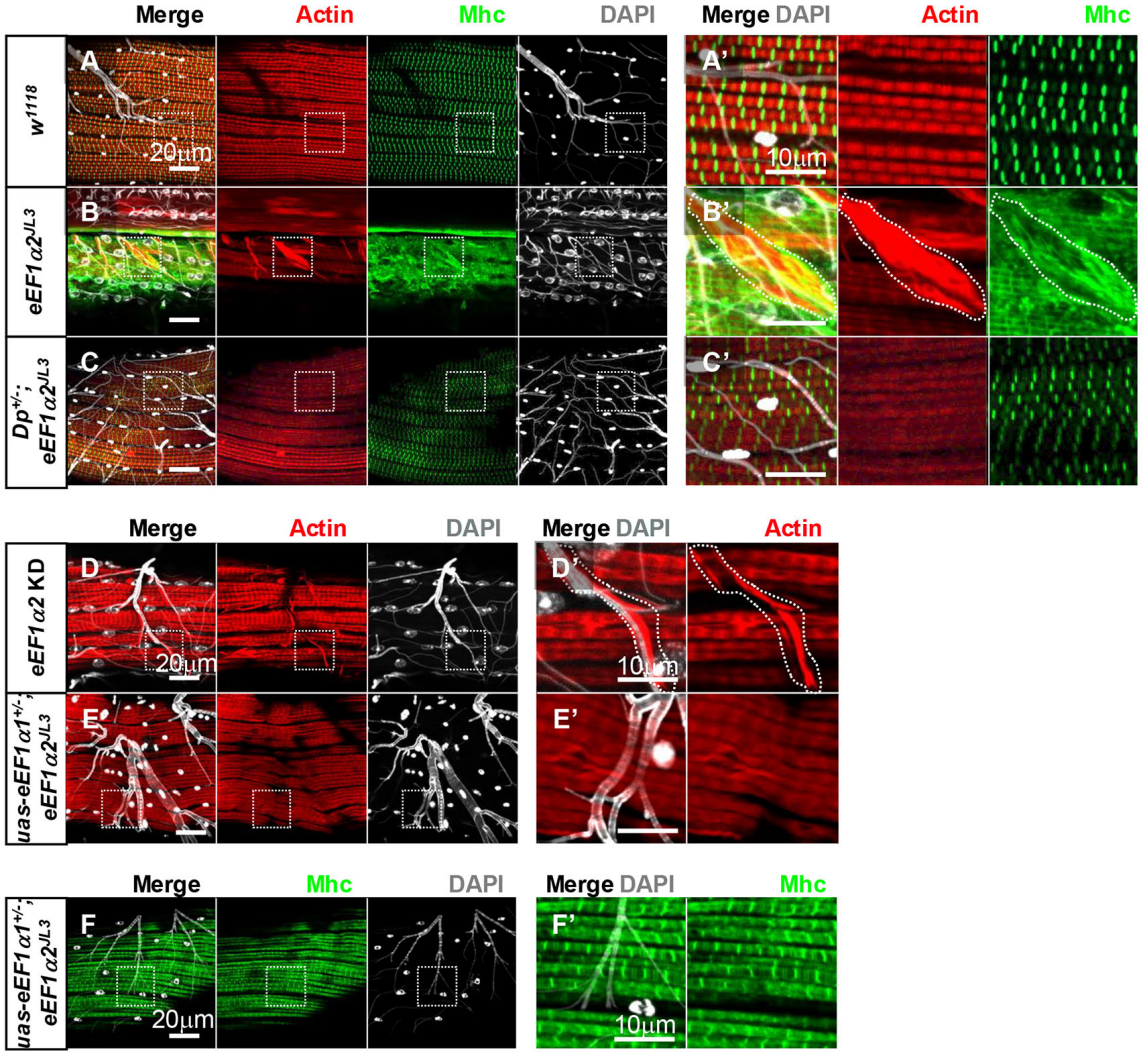
**Ectopic actin and myosin distributions around the peripheral trachea of flies over 30 days old.** (A-Fʹ) Actin (red), Mhc (green) and DAPI (grayscale) staining in IFMs of flies more than 30 days old. Genotypes: control *w^1118^* (A,A′), *eEF1α2^JL3^* homozygous mutants (B,B′), *Dp^+/−^;eEF1α2^JL3^* (C,C′), IFM-specific *eEF1α2* knockdown (KD) (D,D′), *eEF1α1* overexpression in *eEF1α2^JL3^* homozygous mutants (E-F′). White dashed squares in A-F are enlarged in Aʹ-Fʹ. White dashed regions mark an example of trachea-ensheathing actin and myosin distributions. Images are representative of three (A), six (B,F), four (C), 11 (D) and two (E) dissected samples.

In *eEF1α2* mutant flies older than 30 days AE, we found ectopic actin and Mhc distributions wrapping distal ends of trachea (Fig. 6B,B′, dashed line), not seen in *eEF1α2* wild-type control flies (Fig. 6A,A′). This mutant phenotype was rescued in *Dp^+/−^*;*eEF1α2^JL3^* (Fig. 6C,C′). Moreover, tissue-specific *eEF1α2* knockdown mimicked the mutant phenotype (Fig. 6D,D′, dashed line). Intriguingly, we did not find actin signals wrapping the trachea in aged *eEF1α2^JL3^* in flies overexpressing *eEF1α1* (Fig. 6E-F′). These results indicate that *eEF1α1* and *eEF1α2* have shared functions in preventing actin accumulation around trachea but not in other aspects of myofiber maintenance in the aging muscles.

### *eEF1α2* loss causes light-dependent photoreceptor degeneration

Recent studies indicate that eEF1α2 is required for neuronal functions that include synaptogenesis (Mendoza et al., 2021; Mohamed and Klann, 2023). Moreover, eEF1α2 mutant mice or worms reportedly suffer from neurodegeneration phenotypes (Newbery et al., 2005; Chalorak et al., 2020). Our own experiments with the fly brain failed to detect increased apoptosis in *eEF1α2* homozygous mutants as assessed through terminal deoxynucleotidyl transferase dUTP nick end labeling (TUNEL) (Fig. S4A-K, yellow arrowheads). Neither did we find significant difference in the levels of two neuronal proteins: Embryonic lethal abnormal vision (Elav), a protein specifically expressed in all neurons, and Cyclic-AMP response element-binding protein B (CREB, encoded by *CrebB*), a transcription factor involved in memory formation (Fig. S4L,M).

**Fig. 7.**
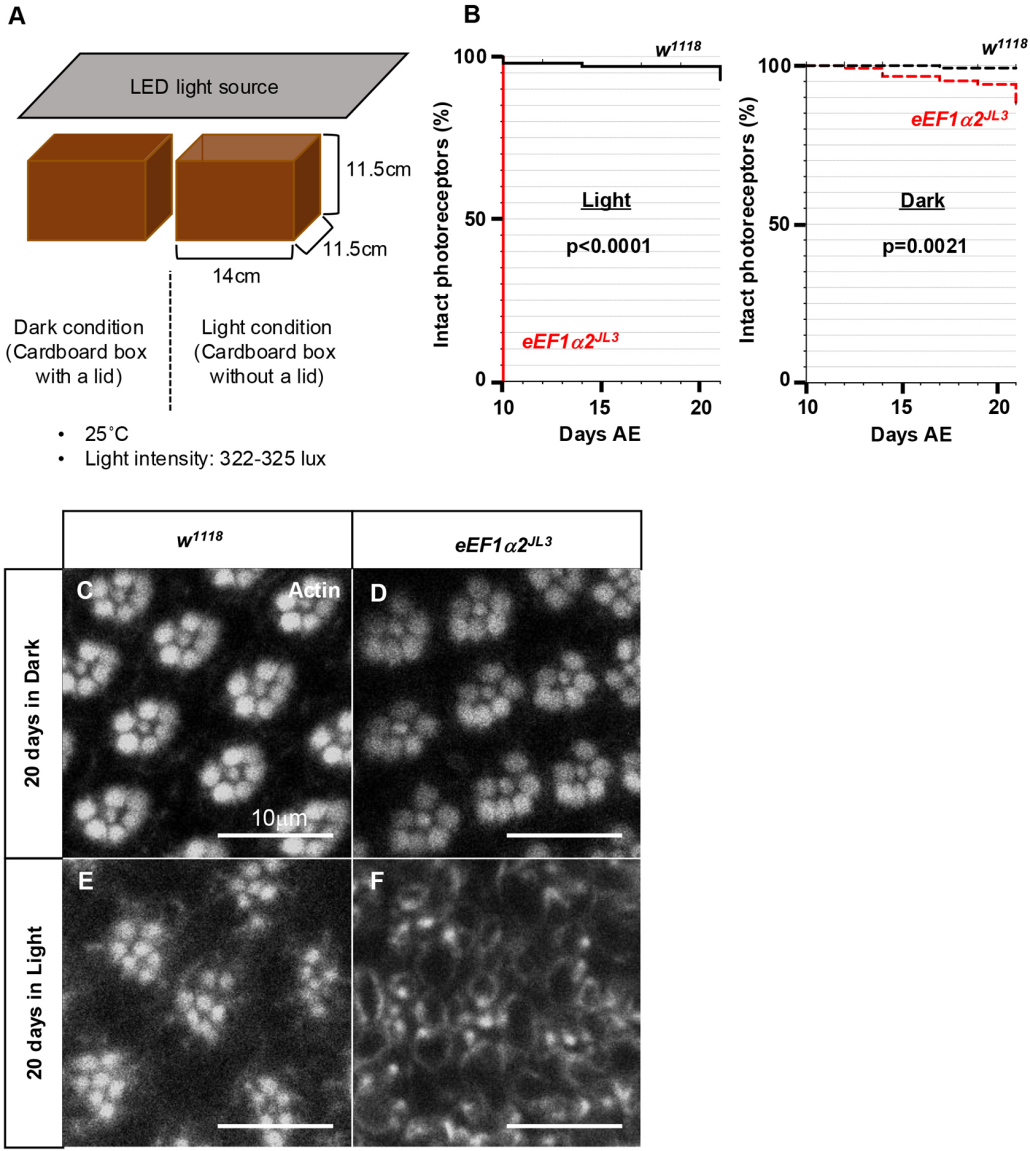
**Light induced-photoreceptor degeneration in *eEF1α2^JL3^* homozygous flies.** (A) Schematic diagram of the experimental setup. The flies were placed in cardboard boxes with or without lids, on top of which was an LED light source. (B) The course of age-related retinal degeneration in live flies assessed through deep pseudopupil assays. Genotypes: control *w^1118^* (solid or dashed black line), *eEF1α2^JL3^* (solid or dashed red line). Note that light exposure caused deep pseudopupil loss specifically in *eEF1α2^JL3^* homozygous flies. *P*-values were calculated by log-rank (Mantel–Cox) test. (C-F) Visualization of photoreceptors from 20-day-old flies reared in the dark (C,D) or under light (E,F), labeled with phalloidin (grayscale). Each photoreceptor contains a rhabdomere that is labeled with phalloidin. A healthy ommatidium contains seven rhabdomeres clustered together in an optical plane, and this pattern is repeated in a regular array throughout the eye. Genotypes: *w^1118^* (C,E), *eEF1α2^JL3^* (D,F). Images are representative of three to five dissected and imaged samples.

For an improved measure of neuronal loss, we turned to the adult *Drosophila* eye, in which precisely eight photoreceptor neurons are arranged within an ommatidium in a specific pattern. We specifically reared wild-type and mutant flies under two different conditions: either under constant light exposure (322-325 lux) or in total darkness for 3 weeks at 25°C (Fig. 7A). To assess the integrity of photoreceptors in live flies over time, we used the deep pseudopupil assay, in which flies with intact photoreceptors reveal psudopupils in response to blue light exposure in live flies (Franceschini and Kirschfeld, 1971; Ostroy et al., 1974; Pichaud and Desplan, 2001; von Lintig et al., 2001). We found that the *eEF1α2^JL3^* mutants showed the loss of deep pseudopupils by 10 days AE when reared under constant light (Fig. 7B). The deep pseudopupil loss did not occur in control flies or when *eEF1α2^JL3^* were reared in the dark (Fig. 7B). To further validate this phenotype, we dissected the *Drosophila* retina and visualized the photoreceptor rhabdomeres with phalloidin labeling (Fig. 7C-F). As reported, wild-type controls showed seven clear photoreceptor rhabdomeres per ommatidium in an optical plane (the eighth photoreceptor is a different optical plane) arranged in a repeating pattern (Fig. 7C,E), but the *eEF1α2^JL3^* ommatidia lost this pattern at 20 days when reared under light (Fig. 7F). *eEF1α2^JL3^* mutants maintained the stereotypical rhabdomere patterns when reared in the dark (Fig. 7D), indicating that *eEF1α2* supports cellular homeostasis against light-induced damages in photoreceptor neurons.

## DISCUSSION

Loss of muscle mass, strength and function significantly contribute to various disabilities associated with old age. Muscle atrophy in humans and *Drosophila* can be impacted by changes in protein synthesis and degradation (Demontis et al., 2013). In addition, factors that regulate actin dynamics are essential for myofiber maintenance (Morales et al., 2023; Clarkson et al., 2004). Among the human diseases associated with actin aggregation in the muscle is a group of congenital diseases known as nemaline myopathies (Wallgren-Pettersson et al., 2011; Nandy et al., 2023). Here, we report evidence that *Drosophila eEF1α2* has a previously unrecognized role in the age-related myofiber homeostasis through an unconventional role.

*Drosophila* eEF1α1 or eEF1α2 are highly conserved proteins, most notable for their roles in delivering aminoacyl-tRNAs to ribosomes for translation. Reflecting its essential role in protein synthesis, mutations in yeast eEF1α cause lethality (Mateyak et al., 2021). At the same time, many studies have reported moonlighting roles of eEF1α beyond its canonical role in mRNA translation elongation. These include the role of *Saccharomyces cerevisiae* eEF1α in inhibiting the kinase GCN2 (Ramesh and Sattlegger, 2020) and its role in actin bundling (Gross and Kinzy, 2005; Silva et al., 2016). In cultured mammalian neurons, eEF1α2 has reported roles in actin assembly and synapse formation (Mendoza et al., 2021). Supporting this finding, classical eEF1α2 mutant mice, referred to as *wasted* (*wst*), suffer from a short lifespan, neuromuscular junction neurodegeneration (Newbery et al., 2005) and reduction of limb strength (Griffiths et al., 2012). However, the short lifespan or lethality has made it difficult to study possible roles of eEF1α2 in age-related cellular homeostasis (Chambers et al., 1998; Schnorrer et al., 2010). We believe that our work on the *Drosophila eEF1α2* CRISPR mutants fills this conceptual gap. We find that *eEF1α2* loss causes at least two distinct age-specific phenotypes. In young flies, the loss of *eEF1α2* results in thinner myofibrils, which could be rescued by the overexpression of its paralog of *eEF1α1.* In old *eEF1α2^JL3^* mutant muscles, actin starts localizing in an uneven pattern from around 10 days AE, further worsening by 30 days AE. This phenotype is not rescued by extra *eEF1α1* expression, indicative of a unique role of *eEF1α2* in this age-related cellular homeostasis.

At the mechanistic level, how could *eEF1α2* regulate muscle homeostasis during aging? An obvious possible cause is the reduction of overall protein synthesis caused by *eEF1α2* loss, which may indirectly impact various cellular homeostatic mechanisms. However, several data argue against this idea. First, we did not see significant changes in the levels of actin and Mhc proteins up to a month after eclosion. Moreover, overexpression of *eEF1α1* failed to rescue the actin distribution phenotype in aged IFMs. Taken together, these observations suggest that myofiber maintenance by *eEF1α2* is likely due to an unconventional role of this gene, neither related to protein synthesis nor shared with *eEF1α1*.

What could be the relevant unconventional function of *eEF1α2*? We note that previous studies have reported direct interactions between eEF1α2 and actin dynamics in *S. cerevisiae* (Gross and Kinzy, 2005), *Tetrahymena* (Morita et al., 2008) and mammal hippocampal neurons (Mendoza et al., 2021). Interestingly, we found that *eEF1α2* loss impacts actomyosin distribution, specifically in older fly muscles. These observations lead us to propose that unconventional functions of *eEF1α2* play particularly essential roles in maintaining cellular homeostasis in aging metazoan tissues.

In summary, this study uncovers a new link between aging, actin dynamics and *eEF1α2*. Loss of muscle homeostasis is a defining feature associated with aging, and the moonlighting role of eEF1α2 appears to be critical against age-related muscular atrophy in *Drosophila*.

## MATERIALS AND METHODS

### Fly genetics

Flies were maintained on regular cornmeal agar medium in a 25°C incubator, and the eclosed flies were moved to fresh food vials. Flies were flipped to new food vials every other day during the aging analysis. The fly strains used in this study are listed in Table S1. To generate *uas-eEF1α1*, *Drosophila eEF1α1* cDNA (UFO01269, *Drosophila* Genomics Resource Center stock 1621179; https://dgrc.bio.indiana.edu//stock/1621179; RRID:DGRC_1621179) was amplified by PCR and subcloned into the pUAST-attB plasmid (*Drosophila* Genomics Resource Center stock 1419). A purified pUAST-*eEF1α1-*attB plasmid was injected to the VK01 strain (9722, Bloomington *Drosophila* Stock Center, IN, USA) through the service of BestGene (CA, USA).

### Generation of the *eEF1α2* mutant allele

*eEF1α2^JL3^* was generated by crossing the ubiquitous sgRNA-expressing strain (GS04210, FBti0206465; Hu et al., 2017) to a line that expresses Cas9 in germ cells (FBti0159183; Bloomington *Drosophila* Stock Center, 54591). The *eEF1α2* mutant sequence was amplified using the primers 5′-ACCACGATGACACGACCC-3′ and 5′-CCAGGGGCATCGATGATGG-3′, and the amplified sequences were purified using the MinElute Gel Extraction Kit (28604, Qiagen). The PCR products were sent to Genewiz for sequence analysis.

### Analysis of wing posture phenotypes

Flies were kept in regular cornmeal vials in a 25°C incubator for 10 days, and the percentage of flies with abnormal wing postures was calculated. Three to four independent groups of flies were assessed for each genotype (*w^1118^*: vial 1, *n*=40; vial 2, *n*=40; vial 3, *n*=31; vial 4, *n*=32. *eEF1α2^JL3^*: vial 1, *n*=40; vial 2, *n*=40; vial 3, *n*=39. *Dp^+/−^;eEF1α2^JL3^*: vial 1, *n*=38; vial 2, *n*=39; vial 3, *n*=56). The statistical significance was assessed using unpaired two-tailed *t*-tests between each genotype.

### Analysis of eclosion rate and body weight in adult flies

To assess the rate of eclosion, more than 30 first instar larvae were collected and cultured on the regular cornmeal agar medium in a 25°C incubator until eclosion. In this assay, four individual groups were prepared for each genotype (*w^1118^*: vial 1, *n*=31; vial 2, *n*=41; vial 3, *n*=41; vial 3, *n*=43. *eEF1α2^JL3^*: vial 1, *n*=40; vial 2, *n*=37; vial 3, *n*=41; vial 3, *n*=41). Then, the number of eclosing flies was counted from day 9 of first instar larvae collection. The plots on the graph are average values of results obtained from four independent vials. The log-rank test was used to assess statistical significance. For body weight analysis, 0- to 1-day-AE flies were collected from each genotype. More than 25 flies were pooled in each vial (*w^1118^*: vial 1, *n*=30; vial 2, *n*=29; vial 3, *n*=30. *eEF1α2^JL3^*: vial 1, *n*=25; vial 2, *n*=30, vial 3, *n*=30). At day 3 after fly collection, flies were transferred to 1.5 ml tubes for weight measurement. The average weight was calculated and the statistical significance was assessed using unpaired two-tailed *t*-test.

### Immunohistochemistry and imaging

Adult IFMs, eyes and larval muscles were dissected in ice-cold phosphate buffered saline (PBS, pH 7.4) and fixed in 4% paraformaldehyde (043368.9M, Thermo Scientific Chemicals) diluted in PBS containing 0.2% Triton X-100 (T8787, Sigma-Aldrich) (PBS-T) for 30 min at room temperature. A standard immunolabeling protocol was followed for subsequent steps. To visualize F-actin, fixed tissues were incubated with Alexa Fluor 594 Phalloidin (1/400, A12381, Invitrogen) for 1 h at room temperature. After washing with PBS-T, the samples were incubated in a 50% glycerol (G33-1, Thermo Fisher Scientific) solution (in PBS) overnight at 4°C, followed by incubation with an 80% glycerol solution (in PBS) containing DAPI (final concentration 60 nM, D9542-1MG, Millipore Sigma) overnight at 4°C. All immunolabeling was done with antibodies diluted in the blocking buffer, consisting of 10% normal goat serum (005-000-121, Jackson ImmunoResearch) diluted in PBS-T. Mhc staining was performed after actin staining. Briefly, phalloidin-stained IFMs were incubated in the blocking buffer for 1 h at room temperature. Then, samples were incubated with a mouse anti-Mhc antibody [1/500, 3E8-3D3, Developmental Studies Hybridoma Bank (DSHB)] overnight at 4°C. Alexa Fluor 488-conjugated goat anti-mouse IgG (1/200, A11001, Invitrogen) was used as a secondary antibody. *Z*-stack images were taken 15-20 μm from the surface of the tissues using Zeiss 700 and 800 microscopes (Oberkochen, Germany). Representative single optical layer images were selected for presentation. Fiji (Schindelin et al., 2019) was used for image analysis. Whole-fly images were taken with a SMZ1500 (Nikon, Tokyo, Japan) microscope and NIS Elements BR camera (Nikon).

### Myofibril measurement

Myofibrils were stained with phalloidin for the measurements. We chose five continuous sarcomeres from five different myofibrils and measured the myofibril thickness at the Z-line using Fiji. *w^1118^*: *n*=125; *eEF1α2^JL3^*: *n*=150; *Dp^+/−^;eEF1α2^JL3^*: *n*=150; *uas-eEF1α1^+/−^;eEF1α2^JL3^*: *n*=150; *dpp^d-Ho^*: *n*=150; where *n* is the number of sarcomeres examined. *P*-values were calculated by unpaired two-tailed *t*-test. Error bars show mean±s.e.m. The analysis was done with samples from three to five individual flies. The data were analyzed on Prism 10 (GraphPad Software, MA, USA).

### Western blot analysis

Adult IFMs were collected from five flies of each genotype and ten whole-fly heads were prepared for each western blot analysis. Each sample set was triplicated. Dissection was carried out in ice-cold PBS. The dissected IFMs and heads were kept on dry ice while other samples were being dissected. All dissected IFMs and heads were stored at −80°C until western blot analysis. The homogenate was prepared using 50 µl of RIPA buffer (150 mM sodium chloride, 1.0% Triton X-100, 0.5% sodium deoxycholate, 0.1% sodium dodecyl sulfate, 50 mM Tris, pH 8.0) containing a protease inhibitor cocktail (11873580001, Roche). 10 µl of homogenates from each sample were run (100 V, 100 min) in SDS gel (5% stacking gel, 10% running gel). Proteins were then transferred (80 V, 60 min) to PVDF membranes (ISEQ00010, Millipore). The membranes were blocked with 10% skim milk dissolved in TBS (pH 7.6) containing 0.1% Tween 20 (TBS-T) for 1 h at room temperature. Then, the membranes were incubated with primary antibodies diluted in TBS-T overnight at 4°C. The following antibodies were used: mouse anti-actin antibody (1/5000, MAB1501, Millipore Sigma), mouse anti-GAPDH antibody (1/1000, sc-365062, Santa Cruz Biotechnology), mouse anti-Mhc antibody (1/10,000, 3E8-3D3-s, DSHB), rat anti-Elav antibody (1/1000, 7E8A10, DSHB) and rabbit anti-CREB antibody (1/1000, 9197T, Cell Signaling Technology). Primary antibodies were visualized with goat anti-mouse, anti-rabbit and anti-rat IgG antibodies by incubation at room temperature for 2 h (1/1000; IRDye 680RD goat anti-mouse IgG secondary antibody, 926-68070; IRDye 800CW goat anti-mouse IgG secondary antibody, 926-32210; IRDye 800CW goat anti-rabbit IgG secondary antibody, 926-32211; IRDye 680RD goat anti-rat IgG secondary antibody, 926-68076; LI-COR). The membrane images were acquired using Odyssey Classic Infrared Imaging System (LI-COR) and analyzed with Fiji. The quantification was analyzed using unpaired two-tailed *t*-test.

### Climbing assay

For the climbing assay, 0- to 1-day-AE flies were collected from each genotype, with more than 20 flies per each vial. Each genotype was assessed through three independent vials of flies (*w^1118^*: vial 1, *n*=28; vial 2, *n*=30; vial 3, *n*=29. *eEF1α2^JL3^*: vial 1, *n*=20; vial 2, *n*=25; vial 3, *n*=28). 20-day-AE flies were subjected to climbing assays. Specifically, we counted the flies that reached the 8 cm height mark from the bottom of a vial within 20 s after being tapped down to the bottom, which was recorded using an iPhone camera. The assay was repeated three times, and the average percentage of flies that reached the 8 cm mark was plotted on the graph. The statistical significance was evaluated with unpaired two-tailed *t*-test.

### Photoreceptor degeneration assay

We collected 0- to 1-day-old flies from each genotype and kept them in regular cornmeal vials covered by parafilm with holes for gas exchange. These vials were put into two cardboard boxes (11.5 cm×11.5 cm×14 cm) with or without a lid. The boxes were placed in a 25°C incubator during the assay. For light exposure experiments, we placed an LED light pad (B4 Tracing light box with internal cord+foldable stand, 14.2×10.6 inch light board for tracing, three-level brightness, 8000 lux tracing light pad for children, VKTEKLAB) on the boxes and adjusted the intensity to be 322-325 lux on the no-lid box. The experimental setup is shown in Fig. 7A. The deep pseudopupil in living flies was observed under blue light with a SMZ1500 microscope (Nikon). We followed the pseudopuils from day 10 to day 21 AE.

### TUNEL assay

Zero- to 1-day-AE flies were collected and cultured until 10 days AE (*w^1118^*, *n*=7; *eEF1α2^JL3^*, *n*=6). ApopTag Red *In Situ* Apoptosis Detection kit (S7165, Millipore Sigma) was used to visualize dead cells in fly brains. The staining was performed following the manufacturer's protocol (specifically, the protocol for ‘fluorescent staining of paraffin-embedded tissue’) with some modifications. At step 2 (‘Pretreat tissue’), fixed whole brains were incubated with proteinase K for 20 min at room temperature. At step 3 (‘Apply equilibration buffer’), samples were incubated in the equilibration buffer for 10 min at room temperature. The stained brains were incubated with Vectashield containing DAPI (H-1200-10, Vector Laboratories) before imaging. Statistical significance was assessed with unpaired two-tailed *t*-test.

## Supplementary Material

10.1242/dmm.050729_sup1 Supplementary information
