# Supplementary material for: eEF1α2 is required for actin cytoskeleton homeostasis in the aging muscle
Source: Dis Model Mech. 2024 Aug 29;17(9):dmm050729. doi: 10.1242/dmm.050729 (PMC11381931; doi:10.1242/dmm.050729)
Supplement: Supplementary information [file dmm-17-050729-s1.pdf]

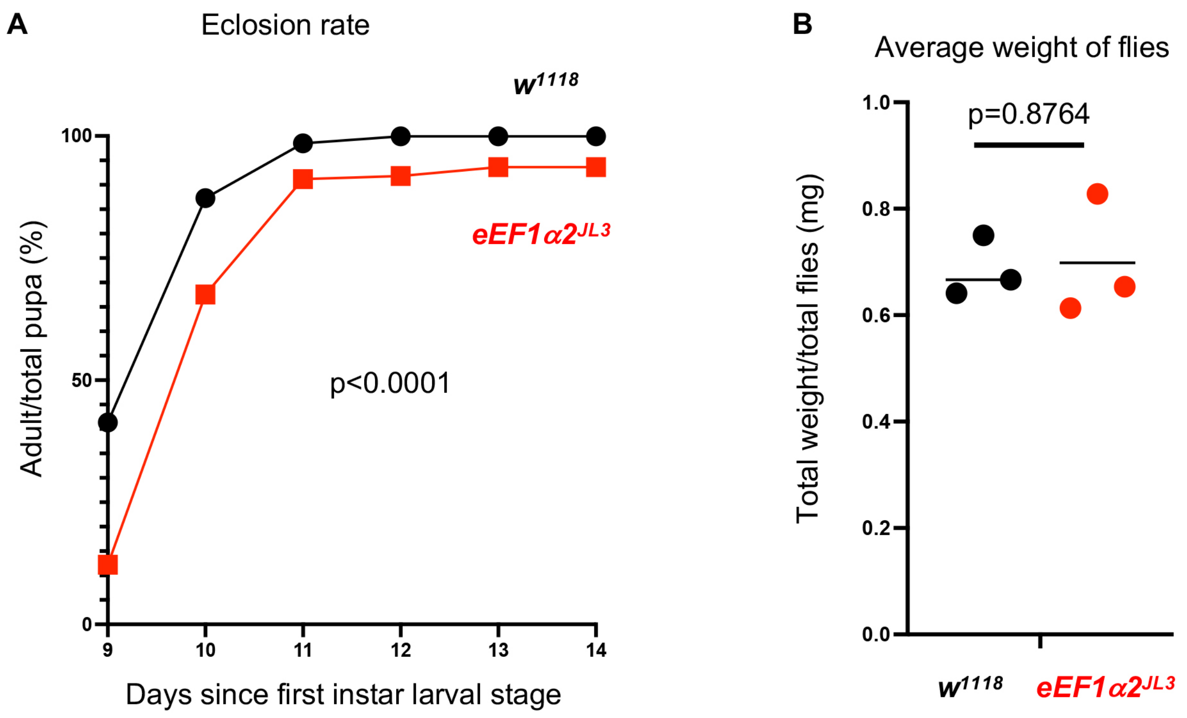

Fig. S1. The mutant developmental rate and body weight.

(A) A graph showing the percentage of eclosing adult flies on indicated dates (x-axis) after first instar larvae collection. Shown are control w^{1118} (black line, n=161) and $eEF1\alpha2^{JL3}$ homozygotes (red line, n=165). Log-rank test was used to assess statistical significance.

(b) Average weight of adult flies of the indicated genotypes. w^{1118}: n=89 (vial1=30, vial2=29, vial3=30), $eEF1\alpha2^{JL3}$: n=85 (vial1=25, vial2=30, vial3=30). Unpaired t test was used to assess statistical significance. The bars show mean values.

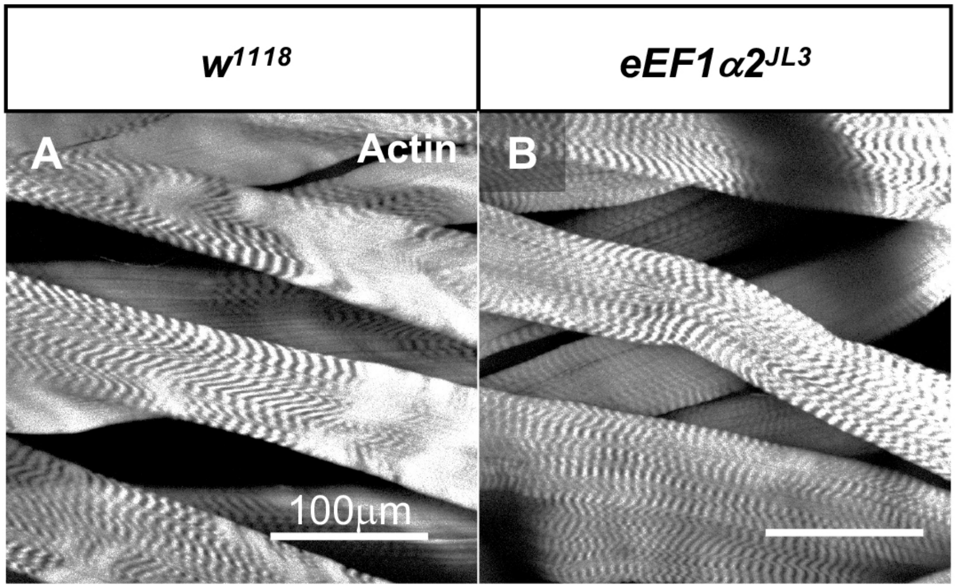

Fig. S2. Actin distribution patterns in third instar larval wall muscles. (A-B) Actin (grayscale, labeled with Phalloidin) distribution in the control w^{1118} larva (A), and $eEF1\alpha2^{JL3}$ (B). Note that both patterns are similar.

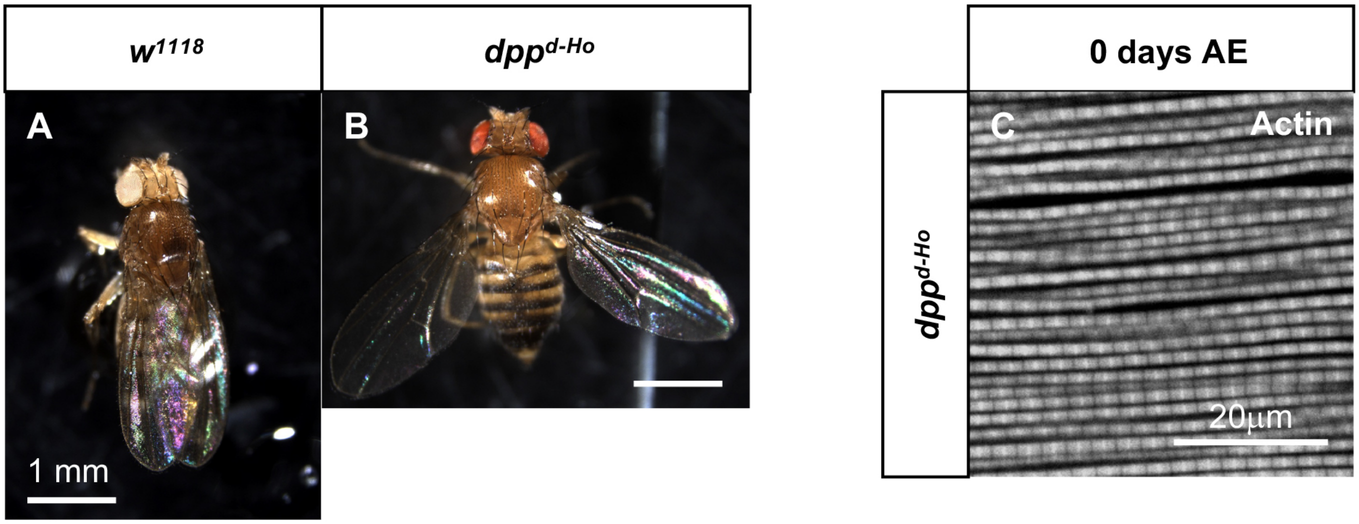

Fig. S3. Phenotypes of the classic wing mutant, *dpp*$^{d\text{-}Ho}$.

(A, B) Whole fly images of control w^{1118} (A) and $dpp^{d\text{-}Ho}$ (B). (C) Actin (grayscale) of IFMs visualized through phalloidin labeling at 0-day AE.

Disease Models & Mechanisms • Supplementary information

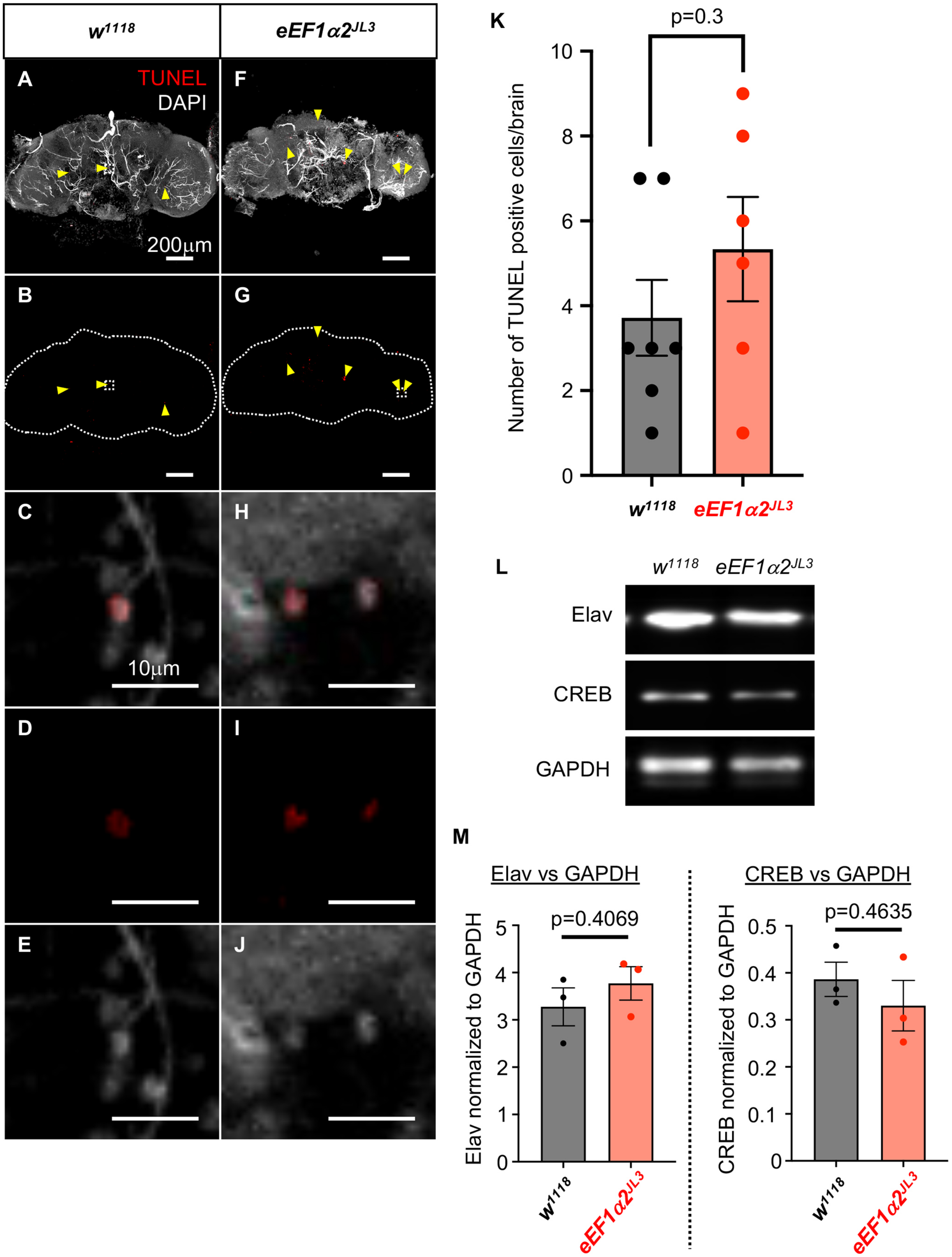

w1118
eEF1α2JL3
A
TUNEL
DAPI
200μm
F
B
G
C
10μm
H
D
I
E
J
K
p=0.3
Number of TUNEL positive cells/brain
0
2
4
6
8
10
w1118
eEF1α2JL3
L
w1118
eEF1α2JL3
Elav
CREB
GAPDH
M
Elav vs GAPDH
p=0.4069
Elav normalized to GAPDH
0
1
2
3
4
5
w1118
eEF1α2JL3
CREB vs GAPDH
p=0.4635
CREB normalized to GAPDH
0
0.1
0.2
0.3
0.4
0.5
w1118
eEF1α2JL3

Fig. S4. TUNEL assay and neuronal marker analysis in adult CNSs of *eEF1α2* mutant.

(A-J) Apoptotic cells (yellow arrowheads) were visualized with TUNEL assays on the brains of 10 days AE w^{1118} and $eEF1\alpha2^{JL3}$ flies. The cell nucleus is stained with TUNEL (red) and/or DAPI (grayscale). Each brain is outlined with white dashed lines. The white dashed squares in (AB, FG) are magnified in (C-E, H-J). (A-E) w^{1118}. (F-J) $eEF1\alpha2^{JL3}$. (K) The nucleus number colocalizing TUNEL and DAPI staining in w^{1118} (n=7) and $eEF1\alpha2^{JL3}$ (n=6) brains. Error bars show SEM, and bar graphs are mean values. P values were calculated with unpaired t tests. (L) Western blots of the indicated proteins using whole brains of 10 days AE w^{1118} and $eEF1\alpha2^{JL3}$. Elav and CREB are neuron-specific proteins. GAPDH serves as a loading control. (M) Quantifications of band intensities in (L). Values of each protein are normalized to GAPDH. Unpaired t test was used to assess statistical significance. Error bars show SEM, and bar graphs are mean values. Whole brains were taken by the *z*-stack imaging, and images were stacked.

Disease Models & Mechanisms • Supplementary information

Table S1. List of fly lines in this paper

Line name	Genetic background	Reference
w^{1118}		Stocked in our lab
nanos-Cas9	*y[1] M{w[+mC]=nanos-Cas9.P}ZH-2A w[*]*	BDSC#54591
eEF1α2 sgRNA	*y[1] sc[*] v[1] sev[21]; P{y[+t7.7] v[+t1.8]=TKO.GS04210}attP40*	BDSC#83495
Act88F-gal4 ;uas-dicer2	*w;Act88F-gal4, act88f-gfp;uas-dicer2/TM6B*	
eEF1α2 KD	*w[1118]; P{GD17776}v52343*	VDRC#52343
VK01	*PBac{yellow[+]-attP-3B}VK00001*	BestGene#9722
uas-eEF1α1	*w*; {uas-eEF1α1}VK00001/CyO*	Ordered to BestGenes
uas-eEF1α1; eEF1α2^{JL3}	*w*; {uas-eEF1α1}VK00001/CyO; eEF1α2*^{JL3}*/TM6B,Tb*	
eEF1α2 duplicated fly	*w[1118]; Dp(3;2)GV-CH321-05C16, PBac{y[+mDint2] w[+mC]=GV-CH321-05C16}VK00037/CyO*	BDSC#90098
eEF1α2 rescued flies	*w*; Dp(3;2)GV-CH321-05C16, PBac{y[+mDint2] w[+mC]=GV-CH321-05C16}VK00037/CyO; eEF1α2*^{JL3}*/TM6B,Tb*	
dpp$^{d\text{-}Ho}$	*dpp[d-ho]*	BDSC#308
Act88F-gal4	*w[*]; P{w[+mC]=Act88F-GAL4.1.3}81B, P{w[+mC]=Act88F:GFP}2/SM6b*	BDSC#38459
Act88F-gal4 ;eEF1α2^{JL3}	*w[*]; P{w[+mC]=Act88F-GAL4.1.3}81B, P{w[+mC]=Act88F:GFP}2/CyO; eEF1α2*^{JL3}*/TM6B,Tb*	
